# Evaluation of a Sample-to-Result POCKIT Central SARS-CoV-2 PCR System

**DOI:** 10.3390/diagnostics13132219

**Published:** 2023-06-29

**Authors:** Jin-Hui Zhu, Chia-Hsing Tai, Chia-Fong Ping, Pin-Hsing Chou, Yun-Long Tsai, Simon Chung, Laura Bradner, Michael Pentella, Phillip Gauger, Jianqiang Zhang

**Affiliations:** 1 Department of Veterinary Diagnostic and Production Animal Medicine, College of Veterinary Medicine, Iowa State University, Ames, IA 50011, USA; miazjh@iastate.edu (J.-H.Z.); lbradner@iastate.edu (L.B.); pcgauger@iastate.edu (P.G.); 2 GeneReach Biotechnology Corporation, Taichung 407, Taiwan; ch.tai@genereachbiotech.com (C.-H.T.); cf.ping@genereachbiotech.com (C.-F.P.); pin@genereachbiotech.com (P.-H.C.); fiwind@gmail.com (Y.-L.T.); simon.chung@genereachbiotech.com (S.C.); 3 State Hygienic Laboratory, University of Iowa, Coralville, IA 52241, USA; michael-pentella@uiowa.edu

**Keywords:** SARS-CoV-2, COVID-19, POCKIT Central, PCR, point-of-care

## Abstract

The emergence of COVID-19 has caused unprecedented impacts on global public health and many other aspects. Meanwhile, many types of methods have been developed to detect the causative agent, SARS-CoV-2; this has greatly advanced the technologies in the diagnostic field. Here, we describe the development and validation of a sample-in-result-out POCKIT Central SARS-CoV-2 PCR system for detecting SARS-CoV-2 in comparison with a commercial reference real-time RT-PCR assay (TaqPath COVID-19 Combo Kit). Both assays were specific and did not cross-react with non-SARS-CoV-2 agents. Both assays were able to detect various SARS-CoV-2 strains including some variants. Based on testing serial dilutions of SARS-CoV-2 USA-WA1/2020 isolate, the limit of detection was 0.8 TCID_50_/mL (1.87 × 10^3^ genomic copies/mL) for POCKIT Central SARS-CoV-2 PCR and 0.16 TCID_50_/mL (3.75 × 10^2^ genomic copies/mL) for the reference PCR. Subsequently, 183 clinical samples were tested by both assays and the diagnostic sensitivity, specificity, and agreement of the POCKIT Central SARS-CoV-2 PCR were 91.7%, 100%, and 94.0%, respectively, when compared to the reference PCR. The compact sample-to-result POCKIT Central SARS-CoV-2 PCR system is a simplified and efficient point-of-care tool for SARS-CoV-2 detection. In addition, this platform can be readily adapted to detect other human and animal viruses.

## 1. Introduction

The pandemic of novel coronavirus disease (COVID-19), caused by severe acute respiratory syndrome coronavirus 2 (SARS-CoV-2), has resulted in a global public health crisis and severely affected the global economy [1,2,3]. As of 12 April 2023, according to the World Health Organization (WHO) report, there have been >762 million confirmed cases and roughly 6.9 million deaths caused by COVID-19 globally. The causative agent SARS-CoV-2 is a positive-stranded RNA virus belonging to the genus *Betacoronavirus* of the family *Coronaviridae*. The SARS-CoV-2 genome in the range of 29.8–29.9 kb in length is composed of multiple open reading frames (ORF). These include ORF1a and ORF1b encoding the polyprotein 1a and polyprotein 1ab which are further processed into 16 non-structural proteins (nsp 1–16), ORFs encoding structural proteins spike (S), envelope (E), membrane (M), and nucleocapsid (N), and some ORFs (e.g., ORFs 3a, 3b, 3c, 3d, 6, 7a, 7b, 8, 9b, 9c, and 10) encoding accessory proteins [4,5].

Coronaviruses are well known for high mutation and recombination rates which drive their genetic diversification [6,7]. In the period of over three years (late 2019 to early 2023), the SARS-CoV-2 virus evolved while rapidly spreading in the human population, resulting in the emergence of various virus variants with different characteristics compared to the ancestral/original strains [8]. Different nomenclature systems have been proposed to describe genetically diverse SARS-CoV-2 strains, and some most notable classification systems include GISAID (www.gisaid.org), Nextstrain (clade.nextstrain.org), and Pango lineages [9,10]. The original SARS-CoV-2 strains in early outbreaks included two Pango lineages A and B [9]. However, with the rapid evolution of SARS-CoV-2, a few thousand Pango lineages have been described (cov-lineages.org). For easy and efficient communications, from May 2021, WHO began to use the Greek alphabet to label the key SARS-CoV-2 variants such as the Alpha (B.1.1.7), Beta (B.1.351), Gamma (P.1, i.e., B.1.1.28.1), Delta (B.1.617.2), Kappa (B.1.617.1), Epsilon (B.1.427 and B.1.429), Eta (B.1.525), Iota (B.1.526), Lambda (C.37), Mu (B.1.621), Omicron (B.1.1.529) variants, and so on (www.who.int; accessed on 15 March 2023).

Rapid, sensitive, and specific methods to detect SARS-CoV-2 and identify the infected individuals are critical to better monitor the infection and limit the spread of COVID-19 [11]. Many advances have been made in laboratory testing for SARS-CoV-2 in the past few years. Molecular diagnostic tools (e.g., real-time PCR, droplet-digital PCR [ddPCR], loop-mediated isothermal amplification [LAMP] assays, and genome sequencing approaches, etc.), rapid antigen tests, antibody tests, and various methods in other formats (e.g., microfluidic and/or biosensor methods) have been developed for detecting or confirming SARS-CoV-2 infection [11,12,13,14,15,16,17,18,19]. Nucleic acid extraction followed by real-time RT-PCR conducted in the central laboratory is considered the gold-standard method for SARS-CoV-2 detection. However, the accuracy of this assay relies on trained personnel and it also requires time-consuming laboratory processes with complex and expensive equipment [11,20]. For these reasons, point-of-care (POC) testing is needed in some resource-limited areas and/or in nursing homes or long-term care facilities [20,21].

Insulated isothermal PCR (iiPCR) is a fluorescent hydrolysis probe-based technology that provides isothermal heating at the bottom of special capillary tubes to induce thermal convection and temperature gradient in an insulated environment to drive PCR reaction within a relatively short amount of time [22,23]. The POCKIT Nucleic Acid Analyzer series, which is a user-friendly iiPCR system (GeneReach Biotech), provide various iiPCR assays for detecting different human pathogens, including malaria, Zika virus, Middle East respiratory syndrome coronavirus (MERS-CoV), and dengue virus [24,25,26,27,28]. The new-generation POCKIT Central system automatically completes both nucleic acid extraction and iiPCR amplification and eventually generates a qualitative result. Simply, it is a sample-in–answer-out equipment that is easy to operate and could be a tool for point-of-care testing.

In 2020, due to the urgent need for SARS-CoV-2 PCR testing, the POCKIT Central SARS-CoV-2 PCR was clinically evaluated on 100 oropharyngeal swab samples [29] in order to apply for emergency use authorization from various agencies. However, in Chang et al. paper [29], the POCKIT Central SARS-CoV-2 PCR was not validated for analytical sensitivity (by testing serial dilutions of a SARS-CoV-2 isolate with known concentration) and specificity (by testing against other non-SARS-CoV-2 pathogens) and was not tested for its capability to detect various SARS-CoV-2 variants. In this study, we thoroughly evaluated the accuracy of the POCKIT Central SARS-CoV-2 orf1ab RT-iiPCR assay in comparison with a commercial TaqPath COVID-19 Combo Kit (Thermo Fisher Scientific).

## 2. Materials and Methods

### 2.1. POCKIT Central SARS-CoV-2 Orf1ab RT-iiPCR System

**Figure 1:**
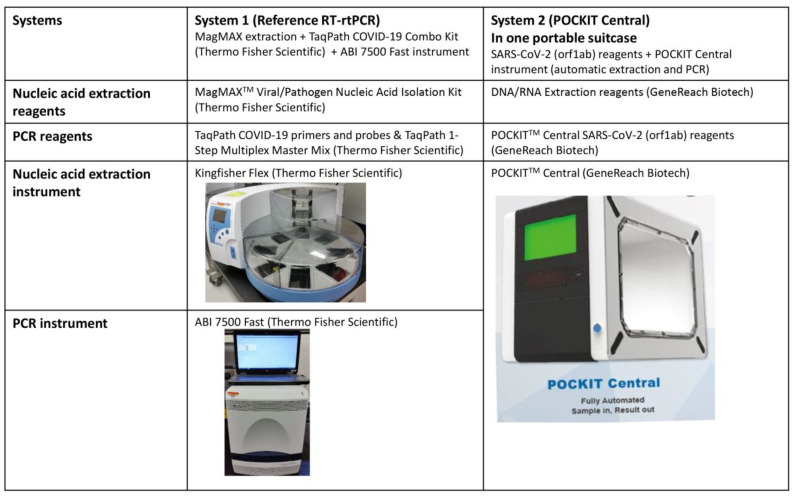
Flow chart of two SARS-CoV-2 PCR systems evaluated in this study.

The POCKIT Central SARS-CoV-2 RT-iiPCR system (GeneReach Biotech, Taichung, Taiwan) used in this study included nucleic acid extraction reagents, SARS-CoV-2 (orf1ab) Premix reagent, and POCKIT Central instrument that allowed to test 8 samples in one run (Figure 1). The PCR assay was designed to target the conserved orf1ab genomic region of SARS-CoV-2 together with a specific Internal Control. The Internal Control template is a plasmid containing a fragment of nucleotides that was artificially designed and synthesized and is not present in any analyzed pathogens or host species. The Internal Control template and the corresponding primers and probe were included in the Premix to monitor the performance of the PCR system. The SARS-CoV-2 primer and probe sequences are: forward primer (ORF1ab-F) 5′-CCCTGTGGGTTTTACACTTAA-3′, reverse primer (ORF1ab-R) 5′-ACGATTGTGCATCAGCTGA-3′, probe (ORF1ab-P) 5′-FAM-CCGTCTGCGGTATGTGGAAAGGTTATGG-BHQ1-3′. The amplicon size is 119 base pairs with the location at nucleotides 13,331–13,449 according to the isolate SARS-CoV-2/human/USA/CA-IGI-0045/2020.

There was one set of Extraction Cartridges, Transfer Cartridges, and SARS-CoV-2 Premix for each sample. Both the nucleic acid extraction and PCR amplification were conducted in one POCKIT Central instrument following the manufacturer’s protocol. Briefly, the Premix vials were snapped into the Transfer Cartridges and placed in the designated slots of the POCKIT Central instrument. Samples (200 µL for each) were loaded into the Extraction Cartridges which were placed in the designated slots of the POCKIT Central instrument. After that, a button was pressed to start the run and it took ~85 min to obtain the results. POCKIT Central SARS-CoV-2 (*orf1ab*) P(+) Control Reagent (GeneReach Biotech, Taichung, Taiwan) was used as an external positive control and a provided Negative Control Reagent was included.

POCKIT Central system automatically completed nucleic acid extraction, iiPCR amplification, and qualitative result interpretation sequentially. Fluorescent signals at 520 nm and 550 nm were generated, respectively, when the targeted SARS-CoV-2 sequences and the internal control sequences were amplified. For results to be valid, the negative control and the SARS-CoV-2 positive control included in the kit must be correct and all of the samples must be Internal Control positive regardless of the SARS-CoV-2 status in the sample.

### 2.2. The Reference TaqPath COVID-19 Real-Time RT-PCR System

In the reference TaqPath COVID-19 real-time RT-PCR system, nucleic acid extraction and PCR reaction were conducted in two separate steps (Figure 1).

A MagMAX Viral/Pathogen Nucleic Acid Isolation Kit (Thermo Fisher Scientific, Waltham, MA, USA) and a Kingfisher Flex instrument (Thermo Fisher Scientific, Waltham, MA, USA) were used to extract nucleic acids from viral pathogens and clinical samples. First, 500 μL of Wash Buffer for Wash 1 Plate, 1000 μL of 80% Ethanol for Wash 2 Plate, and 50 μL of Elution Solution for Elution Plate were prepared per well. Then the Binding Bead Mix with 265 μL of Binding Solution and 10 μL of Total Nucleic Acid Magnetic Beads per reaction was prepared. Next, the sample plate was prepared with 5 μL of Proteinase K, 200 μL of sample, and 275 μL of the Binding Bead Mix per well. Subsequently, 5 μL of MS2 Phage Control (Thermo Fisher Scientific) was added to each sample well and the Negative Control well. Eventually, all of the prepared plates were loaded into a Kingfisher Flex instrument and the automated program was run to extract nucleic acids. The extracted nucleic acids were immediately used for TaqPath COVID-19 PCR testing. The remaining nucleic acids were saved at −80 °C freezer.

TaqPath COVID-19 Combo Kit (Thermo Fisher Scientific) was included as the reference PCR assay for evaluation and comparison in this study. For PCR reaction setup, 6.25 µL of TaqPath 1-Step Multiplex Master Mix (No ROX) (4×), 1.25 µL of COVID-19 Real-Time PCR Assay Multiplex, 7.50 µL of nuclease-free water and 10.0 µL of the RNA template were included in a 25 µL reaction on the 96-well reaction plate. The Positive Control of this assay was TaqPath COVID-19 Control (1 × 10^4^ copies/μL) diluted to a working stock of 25 copies/μL provided by the manufacturer. The Negative Control was included for each RT-PCR reaction plate. PCR reactions were performed on the ABI 7500 Fast instrument (Thermo Fisher Scientific) with the following conditions: one cycle of 25 °C for 2 min, one cycle of 53 °C for 10 min, one cycle of 95 °C for 2 min, and 40 cycles of 95 °C for 3 s and 60 °C for 30 s. COVID-19 Interpretive Software 1.5 version was used for analysis. The reference TaqPath COVID-19 assay included primers and probes targeting three SARS-CoV-2 genes (ORF1ab, N, and S) and one internal positive control MS2. For each target, Ct < 37 was considered Positive and Ct ≥ 37 was considered Negative. For the result interpretation, if ≥2 SARS-CoV-2 targets were positive and MS2 was either positive or negative, the sample was considered Positive; if one SARS-CoV-2 target gene was positive and MS2 was either positive or negative, the sample was considered Inconclusive and would be retested; if 3 SARS-CoV-2 targets were all negative and MS2 was positive, the sample was considered Not Detected for SARS-CoV-2; if 3 SARS-CoV-2 targets and MS2 were all negative, the result was Invalid and retest was required.

### 2.3. Viral and Bacterial Pathogens

To evaluate the cross-reactivity (analytical specificity) of the POCKIT Central SARS-CoV-2 PCR assay and the reference TaqPath COVID-19 assay, a variety of human viral and bacterial pathogens were included for testing (Table 1).

To evaluate the inclusivity of the assays, different SARS-CoV-2 original strains, and variants were tested. These 22 SARS-CoV-2 original strains and variants were obtained from BEI Resources in the format of either genomic RNA or heat-inactivated virus (Table 2). For the heat-inactivated virus strains, 200 µL of the virus was used in the sample-in-result-out POCKIT Central SARS-CoV-2 PCR system; 200 µL of the virus was used for nucleic acid extraction followed by PCR in the TaqPath COVID-19 PCR system. For SARS-CoV genomic RNA obtained from BEI Resources, 10 µL of the genomic RNA was diluted into 190 µL of nuclease-free water and then used in the POCKIT Central SARS-CoV-2 PCR system while 10 µL of the genomic RNA was directly used for TaqPath COVID-19 PCR reaction without going through nucleic acid extraction.

The whole genome sequences of these 22 SARS-CoV-2 strains were obtained from GenBank or GISAID. Then, the 22 whole genome sequences were used as the query sequences at the websites clades.nextstrain.org and gisaid.org to determine the Pango lineage, Nextstrain clade, and GISAID clade information of these strains. In addition, the ORF1ab sequences of 23 SARS-CoV-2 strains (22 strains included in this study and the primary strain Wuhan/WIV04/2019) were aligned and compared using BioEdit software (version 7.2.5) to determine the conservation of POCKIT Central SARS-CoV-2 PCR primer and probe sequences.

### 2.4. Clinical Samples

To evaluate the diagnostic accuracy of the POCKIT Central SARS-CoV-2 PCR in comparison with the reference TaqPath COVID-19 PCR, a total of 183 human nasopharyngeal swab samples collected and submitted to the State Hygienic Laboratory of University of Iowa or the Iowa State University Public Health Testing Services in 2020 and 2021 were tested. Among the 183 samples, 100 of them were collected in 2020 and the remaining 83 samples were collected in 2021; all of the samples were collected in the USA.

### 2.5. Limit of Detection of POCKIT Central SARS-CoV-2 PCR Assay and the Reference TaqPath COVID-19 PCR Assay

The heat-inactivated SARS-CoV-2 USA-WA1/2020 isolate (BEI Resources Cat#: NR-52286) with a titer of 1.6 × 10^5^ TCID_50_/mL before heat-inactivation and 3.75 × 10^8^ genomic copies (GC)/mL determined by digital PCR at BEI Resources were serially diluted and used to determine the limit of detection (LOD) of the POCKIT Central SARS-CoV-2 PCR and the reference PCR with 5 replicates at high concentrations and 20 replicates at low concentrations. For the reference PCR, the serially diluted isolate was first extracted with 200 µL input for each dilution and eluted into 50 µL, and then 10 µL of the extracted RNA was used for PCR setup. For the POCKIT Central SARS-CoV-2 PCR, 200 µL of the sample at each dilution was directly used in the system in which nucleic acid extraction and PCR reaction were combined in one instrument.

## 3. Results

### 3.1. Analytical Specificity of POCKIT Central SARS-CoV-2 orf1ab RT-iiPCR Assay and the Reference TaqPath COVID-19 PCR Assay

As shown in Table 1, both POCKIT Central SARS-CoV-2 PCR and the reference PCR had great analytical specificity and did not cross-react with any of the tested non-SARS-CoV-2 viral or bacterial pathogens.

### 3.2. Inclusivity of POCKIT Central SARS-CoV-2 orf1ab RT-iiPCR Assay and the Reference TaqPath COVID-19 PCR Assay

**Figure 2:**
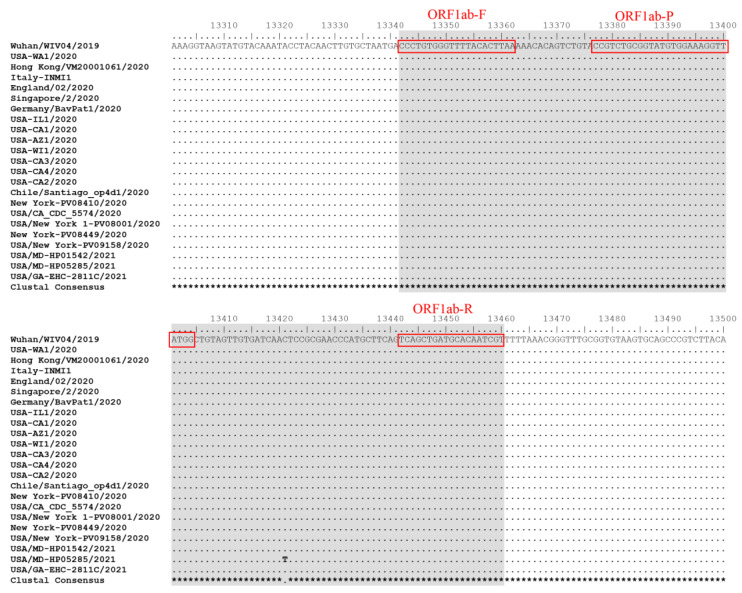
Nucleotide sequence alignment of 23 SARS-CoV-2 ORF1ab genomic regions covering the primers and probe of POCKIT Central SARS-CoV-2 orf1ab RT-iiPCR. The fragment amplified by POCKIT Central SARS-CoV-2 orf1ab RT-iiPCR is highlighted in gray color and the sequences of primers and probes are shown in red boxes.

A total of 22 different SARS-CoV-2 isolates obtained from BEI Resources were tested by both PCR assays. These 22 isolates include the original SARS-CoV-2 strains detected in various countries and regions as well as the SARS-CoV-2 Alpha, Beta, Delta, and Omicron variants. We also determined the Pango lineage, Nextstrain clade, and GISAID clade information of these SARS-CoV-2 isolates with the data summarized in Table 2. All of the 22 isolates tested positive by both PCR assays (Table 2), suggesting that both PCR assays had good coverage to detect genetically diverse SARS-CoV-2 strains. Sequence alignment indicated that the ORF1ab genomic region targeted by POCKIT Central SARS-CoV-2 PCR primers and probe was conserved among all of the evaluated SARS-CoV-2 strains except one SARS-CoV-2 isolate USA/MD-HP05285/2021 which had one nucleotide substitution compared to other SARS-CoV-2 isolates (Figure 2). However, this nucleotide substitution did not affect the detection of USA/MD-HP05285/2021 isolate by POCKIT Central SARS-CoV-2 PCR.

### 3.3. Limit of Detection of POCKIT Central SARS-CoV-2 orf1ab RT-iiPCR Assay and the Reference TaqPath COVID-19 PCR Assay

Different dilutions of the SARS-CoV-2 isolate USA-WA1/2020 were tested by both the POCKIT Central SARS-CoV-2 PCR and the reference PCR, with 5 replicates at high concentrations and 20 replicates at low concentrations. As shown in Table 3, the limit of detection (at least 95% of reactions were positive) of the POCKIT Central SARS-CoV-2 PCR was 1.87 × 10^3^ genomic copies/mL (corresponding to 0.8 TCID_50_/mL) while the limit of detection of the reference PCR was 3.75 × 10^2^ genomic copies/mL (corresponding to 0.16 TCID_50_/mL) under the conditions of this study.

### 3.4. Diagnostic Accuracy of POCKIT Central SARS-CoV-2 orf1ab RT-iiPCR Assay

The diagnostic accuracy of the POCKIT Central SARS-CoV-2 PCR was evaluated by testing 183 clinical samples in comparison with the reference TaqPath COVID-19 PCR. The detailed results are shown in Supplemental Table S1. For all 183 samples, the Internal Control of the POCKIT Central SARS-CoV-2 PCR and the internal positive control (MS2 gene) of the reference PCR were all positive; hence, all of the SARS-CoV-2 results of the two PCR assays were valid.

Regarding the reference PCR, 51 samples were negative (Ct ≥ 37) by all of the three SARS-CoV-2 target genes ORF1ab, N, and S, 94 samples were positive (Ct < 37) by all of the three target genes, and 38 samples were positive by the ORF1ab and N target genes but negative by the S target gene (Table S1). According to the interpretation criteria from the kit manufacturer, it was concluded that 51 samples were negative and 132 samples were positive for SARS-CoV-2 by the reference PCR. For the 132 samples positive by SARS-CoV-2 ORF1ab and N target genes of the reference PCR, the Ct values ranged from 9.46–33.86 (ORF1ab target gene) and 8.12–36.97 (N target gene). For the 38 samples that were positive by the ORF1ab and N target genes but negative by the S target gene, their Ct values ranged from 10.04–33.86 (ORF1ab target gene) and 8.81–34.20 (N target gene).

Regarding the POCKIT Central SARS-CoV-2 PCR, 62 samples were negative and 121 samples were positive for SARS-CoV-2. Compared with the reference PCR, the diagnostic sensitivity, specificity, and agreement of the POCKIT Central SARS-CoV-2 PCR were 91.7%, 100%, and 94.0%, respectively, with all of the 183 samples being accounted for (Table 4). There were 11 discrepant results among the 183 clinical samples between the two PCR assays and all of these 11 samples were negative by the POCKIT Central SARS-CoV-2 PCR but positive by the reference PCR. These 11 samples had relatively high Ct values of 29.10–33.86 (ORF1ab target gene) and 29.19–36.97 (N target gene) according to the reference PCR. As shown in Table S1, for 113 clinical samples with TaqPath ORF1ab PCR Ct < 29, POCKIT Central SARS-CoV-2 PCR had 100% positive percent agreement (113/113); for 115 clinical samples with TaqPath ORF1ab PCR Ct < 30, POCKIT Central SARS-CoV-2 PCR had 99.1% positive percent agreement (114/115); for 125 clinical samples with TaqPath ORF1ab PCR Ct ≤ 32.50, POCKIT Central SARS-CoV-2 PCR had 96.8% positive percent agreement (121/125).

## 4. Discussion

The COVID-19 pandemic has prompted an unprecedented global effort to develop diagnostic methods, vaccines, antiviral drugs, and bioinformatics tools to track virus evolution and spread; all of these have greatly contributed to controlling SARS-CoV-2 infection worldwide. In addition, the knowledge and experience learned from these efforts are invaluable for fighting against other infectious diseases. The speedy development and application of effective mRNA vaccines against COVID-19 is a stellar example of new technology. Similarly, significant advances have been made in the SARS-CoV-2 diagnostic field. According to the 360D× that tracks the commercially available SARS-CoV-2 tests for diagnostic and clinical use (https://www.360dx.com/coronavirus-test-tracker-launched-COVID-19-tests), up to 15 March 2023, there have been 403 different types of PCR tests, 36 isothermal amplification tests, 102 antigen tests, 155 antibody tests, and a few biosensor tests for SARS-CoV-2 worldwide.

Various real-time RT-PCRs targeting ORF1ab, N, S, and E genomic regions have been developed and widely used for detecting SARS-CoV-2, and are considered the gold standard for SARS-CoV-2 testing [11,12,13,19]. However, at the early stage of the COVID-19 outbreak, human medicine diagnostic laboratories did not have sufficient capacity (shortage of supplies, reagents, and instruments for nucleic acid extraction and real-time RT-PCR) to meet the exploding SARS-CoV-2 PCR testing requests. Many veterinary diagnostic laboratories (including our lab at Iowa State University College of Veterinary Medicine) were brought in to test human samples for SARS-CoV-2 by real-time RT-PCR in a high-throughput format. However, the instrument for such high-throughput SARS-CoV-2 real-time RT-PCR is not field deployable and is unsuitable for point-of-care use.

POCKIT Central insulated isothermal PCR system combines nucleic acid extraction and PCR reaction in one instrument and automates the sample-to-answer process within ~85 min; this system can be a useful POC tool. In this study, we evaluated POCKIT Central SARS-CoV-2 PCR system in comparison with a commercial TaqPath COVID-19 real-time RT-PCR which was used as a reference assay. The POCKIT Central SARS-CoV-2 PCR assay showed great analytical specificity and inclusivity. The primers and probe of POCKIT Central SARS-CoV-2 PCR target the conserved ORF1ab genomic region, enabling this assay to successfully detect all of the 22 SARS-CoV-2 original strains and various variants evaluated in this study (Table 2).

The POCKIT Central SARS-CoV-2 PCR (LOD 1.87 × 10^3^ genomic copies/mL) showed lower analytical sensitivity than the reference TaqPath PCR (LOD 3.75 × 10^2^ genomic copies/mL) based on testing the serial dilutions of heat-inactivated SARS-CoV-2 isolate USA-WA1/2020 (Table 3). For the POCKIT Central SARS-CoV-2 PCR, the equipment automatically processed 200 µL of samples from the beginning to the result interpretation. As for the reference PCR, 200 µL of the samples were first extracted using the MagMAX Viral/Pathogen Nucleic Acid Isolation Kit on a Kingfisher Flex instrument to obtain 50 µL of viral nucleic acids, then 10 µL of the nucleic acid extracts were used to set up the PCR reaction. In order to reasonably compare the LODs of these two PCR assays, we calculated the LODs based on the genomic copies of the SARS-CoV-2 isolate before nucleic acid extraction. We did not calculate the LODs based on the concentration of viral RNA used for the PCR reaction because we could not measure the viral RNA concentration in the POCKIT Central SARS-CoV-2 PCR system due to its automated sample-to-result process. Therefore, it is noteworthy that the LOD of the reference TaqPath COVID-19 PCR determined in this study may be different from the LOD based on the quantitation of viral RNA used for PCR. For example, in this study, if we calculated the LOD in another way, the LOD of the reference TaqPath COVID-19 PCR would be 15 genomic copies per reaction (3.75 × 10^2^ genomic copies/mL × 0.2 mL × 10 µL/50 µL).

In this study, among the 183 clinical samples, 38 samples were positive by the reference TaqPath COVID-19 PCR ORF1ab and N target genes but negative by the reference PCR S target gene (Table S1). But, this is not surprising because the S gene is known to be highly prone to mutation. This also emphasizes that when developing SARS-CoV-2 screening PCR, the S gene should not be the single target for primers and probes. When all of the 183 clinical samples were used to evaluate the diagnostic accuracy, the POCKIT Central SARS-CoV-2 PCR system had 91.7% sensitivity (121/132) and 100% specificity (51/51) compared to the reference TaqPath COVID-19 PCR system (Table 4). The 11 samples that were negative by the POCKIT Central SARS-CoV-2 PCR but positive by the reference PCR had relatively high Ct values of 29.10–33.86 by TaqPath PCR ORF1ab target gene. For clinical samples with lower Ct values by TaqPath ORF1ab PCR, POCKIT Central SARS-CoV-2 PCR had better positive percent agreement (e.g., 100% for Ct < 29, 99.1% for Ct < 30, and 96.8% for Ct ≤ 32.50) (Table S1). In 2020, the POCKIT Central SARS-CoV-2 PCR was clinically evaluated on 100 oropharyngeal swab samples and it had 96.8% diagnostic sensitivity compared to a SARS-CoV-2 real-time RT-PCR assay [29]. Such preliminary data were used to apply for emergency use authorization from various agencies. Specifically, in 2020–2022, POCKIT Central SARS-CoV-2 PCR was successfully registered under Food and Drug Administration for emergency use authorization in Taiwan, under Agência Nacional de Vigilância Sanitária (ANVISA) in Brazil, and under the In Vitro-Diagnostic Medical Devices Directive 98/79/EC (IVDD) in Europe. In our current study, the POCKIT Central SARS-CoV-2 PCR was thoroughly validated and the study can be considered a combination of technical and clinical validations. The data from this study confirms the validity of the authorization to use this test in an emergency setting based on previous incomplete data. This work demonstrates the diagnostic reliability of the test and the data presented in the current study can be used for additional marketing registrations if needed.

According to the literature, various other PCR-based POC devices have also been developed and validated for SARS-CoV-2 testing [19]. For example, when compared with a standard real-time RT-PCR, the Accula SARS-CoV-2 POC PCR had 68% overall positive percent agreement (34/50) but had 100% positive percent agreement (27/27) for samples with Ct < 30 [30]. Similarly, when compared with a standard real-time RT-PCR, the Visby Medical COVID-19 POC PCR had 95% overall positive percent agreement (58/61) but had 100% positive percent agreement (49/49) for samples with Ct < 31 [31]. Many rapid antigen tests have also been developed for SARS-CoV-2 POC testing. In one review study [32], the authors included 93 studies (reported in 88 publications) that evaluated 36 rapid SARS-CoV-2 antigen tests in 104,961 participants and the rapid antigen tests had an overall sensitivity of 75%. In one study, the SARS-CoV-2 antigen test had 78.9% sensitivity among all symptomatic participants but 96.3% sensitivity in the symptomatic participants who had PCR Ct values of <29 [33]. Similarly, other studies showed that SARS-CoV-2 antigen tests had better sensitivity in samples with PCR Ct values of <25 and the sensitivity decreased a lot in samples with Ct values of >30 [17,34]. Hence, if the sensitivities of various assays are simply compared relative to the real-time RT-PCR Ct values, it appears that the POCKIT Central SARS-CoV-2 PCR had at least comparable sensitivity when compared to other point-of-care PCR and antigen tests.

## 5. Conclusions

In this study, we described a novel, automated sample-to-result POCKIT Central SARS-CoV-2 orf1ab RT-iiPCR system that combined nucleic acid extraction and PCR reaction in one instrument but still had comparable diagnostic accuracy to the reference real-time RT-PCR, especially for samples with Ct < 30. The compact POCKIT Central SARS-CoV-2 PCR system can be easily set up and implemented in local clinics, health centers, nursing homes, naval ships, cruises, and remote areas when high-throughput testing of a large number of samples is not needed. This system will allow timely (~85 min from loading samples to obtaining the results) on-site detection without transporting samples to a central diagnostic laboratory. In addition, this platform can be readily adapted to detect other human and animal viruses.

## Figures and Tables

**Table 1:** Analytical specificity of POCKIT Central SARS-CoV-2 RT-iiPCR assay and the reference TaqPath COVID-19 PCR assay.

Pathogen	ATCC or BEI Resources	POCKIT Central SARS-CoV-2 (*orf 1ab*) PCR Result	TaqPath COVID-19 RT-PCR Result
*Pseudomonas aeruginosa*	ATCC 27853	Negative	Negative
*Staphylococcus aureus* subsp. *Aureus* Rosenbach	ATCC 8095	Negative	Negative
Influenza A Virus (H1N1)	ATCC VR-1469	Negative	Negative
Influenza B Virus	ATCC VR-1931	Negative	Negative
SARS coronavirus Urbani strain RNA	BEI NR-52346	Negative	Negative
MERS coronavirus EMC/2012, heat-inactivated	BEI NR-50171	Negative	Negative
Human coronavirus NL63	ATCC VR-3263SD	Negative	Negative
Human coronavirus 229E	ATCC VR-740	Negative	Negative
Human coronavirus OC43	ATCC VR-1558D	Negative	Negative
Human adenovirus 5	ATCC VR-5	Negative	Negative
Respiratory syncytial virus	ATCC VR-1540	Negative	Negative
Human rhinovirus 1A	ATCC VR-1559	Negative	Negative
Human parainfluenza 1	ATCC VR-94	Negative	Negative
Human parainfluenza 2	ATCC VR-92	Negative	Negative
Human parainfluenza 3	ATCC VR-93	Negative	Negative

**Table 2:** Inclusivity of POCKIT Central SARS-CoV-2 RT-iiPCR assay and the reference TaqPath COVID-19 PCR assay for detecting different SARS-CoV-2 strains.

SARS-CoV-2 Strain	Type of Material	BEI Resources	GenBank Accession Number or GISAID EPI_SET ID	WHO Label	Pango Lineage	Next strain Clade	GISAID clade	POCKIT Central SARS-CoV-2 PCR	TaqPath COVID-19 RT-PCR
USA-WA1/2020 virus	Heat-inactivated	NR-52286	MT576653		A	19B	S	Positive	Positive
Hong Kong/VM20001061/2020	Genomic RNA	NR-52388	MT644268		A	19B	S	Positive	Positive
Italy-INMI1	Genomic RNA	NR-52498	MT077125		B	19A	V	Positive	Positive
England/02/2020	Genomic RNA	NR-52499	EPI_ISL_407073		A	19B	S	Positive	Positive
Singapore/2/2020	Genomic RNA	NR-52501	EPI_ISL_407987		B	19A	L	Positive	Positive
Germany/BavPat1/2020	Genomic RNA	NR-52502	MT270101		B.1	20A	G	Positive	Positive
USA-IL1/2020	Genomic RNA	NR-52503	MN988713		B	19A	Other	Positive	Positive
USA-CA1/2020	Genomic RNA	NR-52504	MN994467		A	19B	S	Positive	Positive
USA-AZ1/2020	Genomic RNA	NR-52505	MN997409		A	19B	S	Positive	Positive
USA-WI1/2020	Genomic RNA	NR-52506	MT039887		B	19A	L	Positive	Positive
USA-CA3/2020	Genomic RNA	NR-52507	MT027062		B	19A	L	Positive	Positive
USA-CA4/2020	Genomic RNA	NR-52508	MT027063		B	19A	L	Positive	Positive
USA-CA2/2020	Genomic RNA	NR-52509	MN994468		B	19A	Other	Positive	Positive
Chile/Santiago_op4d1/2020	Genomic RNA	NR-52510	EPI_ISL_415661		A.2	19B	S	Positive	Positive
New Yor-PV08410/2020	Genomic RNA	NR-53518	MT370900		B.1	20C	GH	Positive	Positive
USA/New York 1-PV08001/2020	Genomic RNA	NR-52389	MT370904		B.4	19A	Other	Positive	Positive
New York-PV08449/2020	Genomic RNA	NR-53519	MT370902		B.1.319	20C	GH	Positive	Positive
USA/New York-PV09158/2020	Genomic RNA	NR-53520	MT371034		B.1	20C	GH	Positive	Positive
USA/CA_CDC_5574/2020 virus	Heat-inactivated	NR-55245	EPI_ISL_751801	Alpha variant	B.1.1.7	20I	GRY	Positive	Positive
USA/MD-HP01542/2021 virus	Heat-inactivated	NR-55350	EPI_ISL_890360	Beta variant	B.1.351	20H	GH	Positive	Positive
USA/MD-HP05285/2021 virus	Heat-inactivated	NR-56128	EPI_ISL_2103264	Delta variant	B.1.617.2	21I	GK	Positive	Positive
USA/GA-EHC-2811C/2021 virus	Heat-inactivated	NR-56495	OL744074	Omicron variant	B.1.1.529 / BA.1	21K	GRA	Positive	Positive

Notes: Pango lineage and Nextstrain clade were determined by entering the query sequences at https:clades.nextstrain.org on 27 March 2023. GISAID clade was determined by entering the query sequences at gisaid.org on 27 March 2023.

**Table 3:** Limit of Detection of POCKIT Central SARS-CoV-2 RT-iiPCR assay and the reference TaqPath COVID-19 PCR assay. The detection endpoint of each assay was shown in bold.

Isolate Concentration (TCID_50_/mL)	Isolate Concentration (Genomic Copies/mL)	POCKIT Central SARS-CoV-2 (*orf 1ab*) PCR		TaqPath COVID-19 RT-PCR	
		% (No. of Pos for Target)	% (No. of Pos for Internal Control)	% (No. of Pos for Target)	% (No. of Pos for Internal Control)
**16**	3.75 × 10^4^	100% (5/5)	100% (5/5)	100% (5/5)	100% (5/5)
12	2.81 × 10^4^	100% (5/5)	100% (5/5)	100% (5/5)	100% (5/5)
8	1.87 × 10^4^	100% (5/5)	100% (5/5)	100% (5/5)	100% (5/5)
4	9.37 × 10^3^	100% (5/5)	100% (5/5)	100% (5/5)	100% (5/5)
1.6	3.75 × 10^3^	100% (5/5)	100% (5/5)	100% (5/5)	100% (5/5)
**0.8**	**1.87 × 10^3^**	**100% (20/20)**	**100% (20/20)**	100% (20/20)	100% (20/20)
0.4	9.37 × 10^2^	90% (18/20)	100% (20/20)	100% (20/20)	100% (20/20)
**0.16**	**3.75 × 10^2^**	60% (12/20)	100% (20/20)	**100% (20/20)**	**100% (20/20)**
0.08	1.87 × 10^2^	15% (3/20)	100% (20/20)	90% (18/20)	100% (20/20)
0.04	9.37 × 10^1^			65% (13/20)	100% (20/20)
0.01	2.34 × 10^1^			10% (2/20)	100% (20/20)

**Table 4:** Diagnostic accuracy of POCKIT Central SARS-CoV-2 RT-iiPCR assay in comparison with the reference TaqPath COVID-19 PCR assay.

		TaqPath COVID-19 RT-PCR		Total
		Positive	Negative	
POCKIT Central SARS-CoV-2 (*orf 1ab*) PCR	Positive	121	0	121
	Negative	11	51	62
	Total	132	51	183
Sensitivity 91.7%; specificity 100%; agreement 94.0%				

## Data Availability

The data set(s) supporting the results of this article are included within the article.

## Supplementary Materials

The following supporting information can be downloaded at: https://www.mdpi.com/article/10.3390/diagnostics13132219/s1, Table S1: Clinical samples tested by POCIT Central SARS-CoV-2 orf1ab PCR and the reference TaqPath COVID-19 PCR.
