# Supplementary material for: Evaluation of a Sample-to-Result POCKIT Central SARS-CoV-2 PCR System
Source: Diagnostics (Basel). 2023 Jun 29;13(13):2219. doi: 10.3390/diagnostics13132219 (PMC10340693; doi:10.3390/diagnostics13132219)
Supplement: Supplementary file 1 [file diagnostics-13-02219-s001.zip › diagnostics-2390854-supplementary.pdf]

Table S1. Clinical samples tested by POCIT Central SARS-CoV-2 orf1ab PCR and the reference TaqPath COVID-19 PCR

Sample	POCKIT Central PCR result		Reference TaqPath COVID-19 PCR result				
	SARS-CoV-2	Internal Control	SARS-CoV-2 Result	SARS-CoV-2 ORF1ab Ct	SARS-CoV-2 N gene Ct	SARS-CoV-2 S gene Ct	MS2 Ct
#001	Pos	Pos	Pos	9.46	9.76	10.57	37.23
#002	Pos	Pos	Pos	9.70	8.12	11.00	28.04
#003	Pos	Pos	Pos	9.76	8.66	11.33	24.89
#004	Pos	Pos	Pos	10.04	9.84	≥37	27.17
#005	Pos	Pos	Pos	10.20	8.76	11.99	25.47
#006	Pos	Pos	Pos	10.75	8.81	≥37	28.52
#007	Pos	Pos	Pos	10.80	8.84	11.41	24.96
#008	Pos	Pos	Pos	10.94	9.55	11.57	29.52
#009	Pos	Pos	Pos	11.40	11.28	12.16	24.73
#010	Pos	Pos	Pos	11.53	11.01	≥37	26.61
#011	Pos	Pos	Pos	11.58	8.61	12.23	25.52
#012	Pos	Pos	Pos	11.81	12.42	12.71	37.32
#013	Pos	Pos	Pos	12.15	12.62	13.64	26.50
#014	Pos	Pos	Pos	12.33	10.86	12.96	23.30
#015	Pos	Pos	Pos	12.47	10.64	≥37	25.61
#016	Pos	Pos	Pos	12.60	10.08	13.14	25.01
#017	Pos	Pos	Pos	12.61	11.91	13.07	24.09
#018	Pos	Pos	Pos	12.87	12.14	13.74	24.29
#019	Pos	Pos	Pos	12.94	10.52	13.41	34.09
#020	Pos	Pos	Pos	13.11	11.50	≥37	26.39
#021	Pos	Pos	Pos	13.18	10.96	13.79	24.00
#022	Pos	Pos	Pos	13.62	12.57	14.29	23.31
#023	Pos	Pos	Pos	13.62	13.54	14.66	25.89
#024	Pos	Pos	Pos	13.68	13.87	≥37	25.14
#025	Pos	Pos	Pos	13.97	14.72	14.34	25.98
#026	Pos	Pos	Pos	14.25	14.93	15.25	26.06
#027	Pos	Pos	Pos	14.33	12.20	14.69	23.89
#028	Pos	Pos	Pos	14.41	12.34	15.12	23.97
#029	Pos	Pos	Pos	14.70	13.24	≥37	25.06
#030	Pos	Pos	Pos	14.74	15.22	15.47	26.29
#031	Pos	Pos	Pos	14.75	13.94	≥37	24.67
#032	Pos	Pos	Pos	14.90	14.53	15.44	23.22
#033	Pos	Pos	Pos	14.92	15.47	15.70	26.87
#034	Pos	Pos	Pos	14.92	10.45	14.99	23.14
#035	Pos	Pos	Pos	15.03	13.95	15.85	23.45
#036	Pos	Pos	Pos	15.12	15.42	15.56	26.34
#037	Pos	Pos	Pos	15.19	14.82	15.57	24.46
#038	Pos	Pos	Pos	15.28	14.88	15.40	26.45
#039	Pos	Pos	Pos	15.31	13.79	≥37	23.97
#040	Pos	Pos	Pos	15.50	13.33	≥37	24.52
#041	Pos	Pos	Pos	15.83	14.50	16.45	23.27
#042	Pos	Pos	Pos	15.89	15.56	16.66	25.54

#043	Pos	Pos	Pos	15.93	16.26	16.32	25.50
#044	Pos	Pos	Pos	16.02	15.26	≥37	23.88
#045	Pos	Pos	Pos	16.15	15.67	≥37	23.97
#046	Pos	Pos	Pos	16.18	16.20	≥37	24.07
#047	Pos	Pos	Pos	16.32	17.31	16.94	23.64
#048	Pos	Pos	Pos	16.32	15.73	≥37	23.69
#049	Pos	Pos	Pos	16.42	13.74	17.23	25.78
#050	Pos	Pos	Pos	16.62	14.64	17.35	23.51
#051	Pos	Pos	Pos	16.68	17.19	17.53	25.77
#052	Pos	Pos	Pos	16.69	16.59	16.80	26.65
#053	Pos	Pos	Pos	16.75	17.22	17.08	23.28
#054	Pos	Pos	Pos	17.45	17.71	18.16	24.78
#055	Pos	Pos	Pos	17.45	16.40	≥37	23.67
#056	Pos	Pos	Pos	17.57	16.97	≥37	23.82
#057	Pos	Pos	Pos	17.65	14.89	17.89	23.36
#058	Pos	Pos	Pos	17.83	17.32	18.66	25.34
#059	Pos	Pos	Pos	18.02	18.82	18.44	25.45
#060	Pos	Pos	Pos	18.07	17.67	18.61	23.07
#061	Pos	Pos	Pos	18.28	17.95	≥37	23.62
#062	Pos	Pos	Pos	18.42	18.65	19.27	25.84
#063	Pos	Pos	Pos	18.45	19.24	19.49	34.47
#064	Pos	Pos	Pos	18.46	18.73	19.36	25.93
#065	Pos	Pos	Pos	18.88	18.55	18.86	23.35
#066	Pos	Pos	Pos	18.94	18.79	19.36	23.63
#067	Pos	Pos	Pos	19.17	18.34	≥37	23.40
#068	Pos	Pos	Pos	19.24	19.24	19.86	23.65
#069	Pos	Pos	Pos	19.25	20.68	19.67	25.27
#070	Pos	Pos	Pos	19.44	20.48	19.83	25.89
#071	Pos	Pos	Pos	19.75	20.26	≥37	23.91
#072	Pos	Pos	Pos	19.87	18.96	20.54	25.10
#073	Pos	Pos	Pos	19.90	20.44	20.76	25.68
#074	Pos	Pos	Pos	20.19	19.40	≥37	23.87
#075	Pos	Pos	Pos	20.43	19.56	≥37	23.61
#076	Pos	Pos	Pos	20.50	20.47	≥37	23.89
#077	Pos	Pos	Pos	20.65	18.36	21.34	23.42
#078	Pos	Pos	Pos	20.75	20.90	21.58	26.06
#079	Pos	Pos	Pos	20.76	21.43	21.73	25.89
#080	Pos	Pos	Pos	20.80	21.60	21.59	23.72
#081	Pos	Pos	Pos	20.88	20.32	21.36	23.63
#082	Pos	Pos	Pos	21.09	21.01	21.84	26.27
#083	Pos	Pos	Pos	21.27	21.56	22.20	25.70
#084	Pos	Pos	Pos	22.06	22.66	22.94	25.48
#085	Pos	Pos	Pos	22.29	20.70	22.65	23.53
#086	Pos	Pos	Pos	22.44	21.99	23.34	25.17
#087	Pos	Pos	Pos	22.46	23.35	23.41	26.23
#088	Pos	Pos	Pos	22.79	23.15	≥37	23.76
#089	Pos	Pos	Pos	22.81	22.21	23.60	25.71
#090	Pos	Pos	Pos	22.88	23.46	23.73	25.09
#091	Pos	Pos	Pos	23.25	23.42	≥37	23.91
#092	Pos	Pos	Pos	23.46	24.43	≥37	23.89
#093	Pos	Pos	Pos	23.60	24.63	24.33	25.65

#094	Pos	Pos	Pos	23.92	23.92	25.10	25.89
#095	Pos	Pos	Pos	24.16	24.03	24.66	25.22
#096	Pos	Pos	Pos	24.71	22.02	25.61	25.31
#097	Pos	Pos	Pos	25.64	26.39	≥37	23.99
#098	Pos	Pos	Pos	25.96	26.42	26.89	25.34
#099	Pos	Pos	Pos	26.04	24.54	26.56	25.21
#100	Pos	Pos	Pos	26.11	24.60	26.71	25.91
#101	Pos	Pos	Pos	26.14	24.66	26.56	25.53
#102	Pos	Pos	Pos	26.91	27.64	≥37	23.78
#103	Pos	Pos	Pos	27.22	25.60	27.91	25.43
#104	Pos	Pos	Pos	27.81	25.73	28.83	25.53
#105	Pos	Pos	Pos	28.56	27.21	30.19	26.08
#106	Pos	Pos	Pos	28.64	28.83	29.34	25.65
#107	Pos	Pos	Pos	28.65	29.32	29.51	26.40
#108	Pos	Pos	Pos	28.66	27.28	29.74	25.24
#109	Pos	Pos	Pos	28.71	25.63	29.34	25.67
#110	Pos	Pos	Pos	28.71	28.61	29.58	25.42
#111	Pos	Pos	Pos	28.83	29.25	29.52	25.10
#112	Pos	Pos	Pos	28.88	27.33	29.70	24.70
#113	Pos	Pos	Pos	28.99	28.32	31.75	23.70
#114	Neg	Pos	Pos	29.10	29.19	29.48	26.04
#115	Pos	Pos	Pos	29.83	27.38	30.34	24.78
#116	Neg	Pos	Pos	30.29	29.84	≥37	23.83
#117	Pos	Pos	Pos	30.45	30.38	31.44	25.57
#118	Pos	Pos	Pos	30.58	28.67	31.39	25.72
#119	Pos	Pos	Pos	31.24	30.01	31.73	25.29
#120	Pos	Pos	Pos	31.37	31.68	≥37	23.52
#121	Neg	Pos	Pos	31.44	30.81	≥37	23.76
#122	Neg	Pos	Pos	31.55	32.13	≥37	23.52
#123	Pos	Pos	Pos	31.64	31.51	≥37	23.93
#124	Pos	Pos	Pos	32.23	31.80	≥37	23.72
#125	Pos	Pos	Pos	32.50	32.20	≥37	23.83
#126	Neg	Pos	Pos	32.56	31.67	≥37	23.85
#127	Neg	Pos	Pos	32.57	30.76	32.66	25.49
#128	Neg	Pos	Pos	32.69	30.28	35.86	25.29
#129	Neg	Pos	Pos	33.23	34.20	≥37	24.07
#130	Neg	Pos	Pos	33.52	37.97	35.01	23.84
#131	Neg	Pos	Pos	33.53	32.32	≥37	23.91
#132	Neg	Pos	Pos	33.86	32.27	≥37	23.52
#133	Neg	Pos	Neg	≥37	≥37	≥37	25.49
#134	Neg	Pos	Neg	≥37	≥37	≥37	25.68
#135	Neg	Pos	Neg	≥37	≥37	≥37	25.32
#136	Neg	Pos	Neg	≥37	≥37	≥37	25.45
#137	Neg	Pos	Neg	≥37	≥37	≥37	25.55
#138	Neg	Pos	Neg	≥37	≥37	≥37	26.09
#139	Neg	Pos	Neg	≥37	≥37	≥37	26.07
#140	Neg	Pos	Neg	≥37	≥37	≥37	26.30
#141	Neg	Pos	Neg	≥37	≥37	≥37	25.53
#142	Neg	Pos	Neg	≥37	≥37	≥37	25.55
#143	Neg	Pos	Neg	≥37	≥37	≥37	24.77
#144	Neg	Pos	Neg	≥37	≥37	≥37	24.94

#145	Neg	Pos	Neg	≥37	≥37	≥37	25.02
#146	Neg	Pos	Neg	≥37	≥37	≥37	24.81
#147	Neg	Pos	Neg	≥37	≥37	≥37	25.78
#148	Neg	Pos	Neg	≥37	≥37	≥37	25.67
#149	Neg	Pos	Neg	≥37	≥37	≥37	26.06
#150	Neg	Pos	Neg	≥37	≥37	≥37	26.33
#151	Neg	Pos	Neg	≥37	≥37	≥37	25.50
#152	Neg	Pos	Neg	≥37	≥37	≥37	26.27
#153	Neg	Pos	Neg	≥37	≥37	≥37	25.97
#154	Neg	Pos	Neg	≥37	≥37	≥37	26.03
#155	Neg	Pos	Neg	≥37	≥37	≥37	25.62
#156	Neg	Pos	Neg	≥37	≥37	≥37	25.11
#157	Neg	Pos	Neg	≥37	≥37	≥37	25.41
#158	Neg	Pos	Neg	≥37	≥37	≥37	25.58
#159	Neg	Pos	Neg	≥37	≥37	≥37	25.96
#160	Neg	Pos	Neg	≥37	≥37	≥37	26.24
#161	Neg	Pos	Neg	≥37	≥37	≥37	25.88
#162	Neg	Pos	Neg	≥37	≥37	≥37	25.72
#163	Neg	Pos	Neg	≥37	≥37	≥37	25.99
#164	Neg	Pos	Neg	≥37	≥37	≥37	25.93
#165	Neg	Pos	Neg	≥37	≥37	≥37	25.63
#166	Neg	Pos	Neg	≥37	≥37	≥37	26.21
#167	Neg	Pos	Neg	≥37	≥37	≥37	26.13
#168	Neg	Pos	Neg	≥37	≥37	≥37	24.85
#169	Neg	Pos	Neg	≥37	≥37	≥37	25.27
#170	Neg	Pos	Neg	≥37	≥37	≥37	24.46
#171	Neg	Pos	Neg	≥37	≥37	≥37	25.14
#172	Neg	Pos	Neg	≥37	≥37	≥37	24.39
#173	Neg	Pos	Neg	≥37	≥37	≥37	24.16
#174	Neg	Pos	Neg	≥37	≥37	≥37	23.63
#175	Neg	Pos	Neg	≥37	≥37	≥37	23.91
#176	Neg	Pos	Neg	≥37	≥37	≥37	23.90
#177	Neg	Pos	Neg	≥37	≥37	≥37	23.84
#178	Neg	Pos	Neg	≥37	≥37	≥37	23.51
#179	Neg	Pos	Neg	≥37	≥37	≥37	23.54
#180	Neg	Pos	Neg	≥37	≥37	≥37	23.38
#181	Neg	Pos	Neg	≥37	≥37	≥37	23.49
#182	Neg	Pos	Neg	≥37	≥37	≥37	23.79
#183	Neg	Pos	Neg	≥37	≥37	≥37	23.67

Notes:

1. Samples are listed in the ascending order by the reference PCR ORF1ab Ct values.
2. The 11 samples with discrepant results between two PCR assays are highlighted in gray color.
3. The 38 samples that were positive by the ORF1ab and N target genes but negative by the S target gene of the reference PCR are shown in red color font.
